# Exome Analysis Identified a Novel Mutation in the RBP4 Gene in a Consanguineous Pedigree with Retinal Dystrophy and Developmental Abnormalities

**DOI:** 10.1371/journal.pone.0050205

**Published:** 2012-11-26

**Authors:** Catherine Cukras, Terry Gaasterland, Pauline Lee, Harini V. Gudiseva, Venkata R. M. Chavali, Raghu Pullakhandam, Bruno Maranhao, Lee Edsall, Sandra Soares, G. Bhanuprakash Reddy, Paul A. Sieving, Radha Ayyagari

**Affiliations:** 1 National Eye Institute, NIH, Bethesda, Maryland, United States of America; 2 Scripps Institution of Oceanography and Institute of Genomic Medicine, University of California San Diego, La Jolla, California, United States of America; 3 The Scripps Research Institute, La Jolla, California, United States of America; 4 Shiley Eye Center, University of California San Diego, La Jolla, California, United States of America; 5 National Institute of Nutrition, Hyderabad, AP, India; 6 National Institute of Deafness and Communication Disorders, NIH, Bethesda, Maryland, United States of America; Innsbruck Medical University, Austria

## Abstract

Retinitis Pigmentosa (RP) is a common form of retinal degeneration characterized by photoreceptor degeneration and retinal pigment epithelium (RPE) atrophy causing loss of visual field and acuities. Exome sequencing identified a novel homozygous splice site variant (c.111+1G>A) in the gene encoding retinol binding protein 4 (RBP4). This change segregated with early onset, progressive, and severe autosomal recessive retinitis pigmentosa (arRP) in an eight member consanguineous pedigree of European ancestry. Additionally, one patient exhibited developmental abnormalities including patent ductus arteriosus and chorioretinal and iris colobomas. The second patient developed acne from young age and extending into the 5^th^ decade. Both patients had undetectable levels of RBP4 in the serum suggesting that this mutation led to either mRNA or protein instability resulting in a null phenotype. In addition, the patients exhibited severe vitamin A deficiency, and diminished serum retinol levels. Circulating transthyretin levels were normal. This study identifies the RBP4 splice site change as the cause of RP in this pedigree. The presence of developmental abnormalities and severe acne in patients with retinal degeneration may indicate the involvement of genes that regulate vitamin A absorption, transport and metabolism.

## Introduction

Retinal dystrophies (RD) are characterized by photoreceptor degeneration and RPE atrophy causing loss of visual field and acuities [1], [2]. These degenerations are inherited in autosomal dominant, autosomal recessive, X-linked, mitochondrial and syndromic forms. More than 170 genes have been implicated in causing RD (Retinal Information Network (RetNet), https://sph.uth.tmc.edu/retnet/). Retinitis pigmentosa (RP) is a common form of RD affecting 1 in 4000 individuals in the United States. The clinical features of RP are delayed dark adaptation and progressive loss of sensitivity, loss of peripheral vision at early stages progressing to tunnel vision or in severe cases, to a total loss of vision at later stages. About 60 genes involved in causing RP have been reported (RetNet), of which 35 genes exhibit a recessive mode of inheritance [3], [4]. These 35 genes are estimated to account for 30 to 50% of recessive RP cases [1]. Identifying causative genes for RP has been challenging due to the significant overlap in disease phenotypes and the lack of sufficient information on phenotype-genotype associations.

Recent advances in exome capture and next generation sequencing methodologies offer efficient strategies to identify the genetic basis of inherited conditions in pedigrees that are not suitable for linkage mapping [5], [6]. These strategies can be used for screening genes implicated in RD as well as for identifying novel genes associated with RD and other related diseases [7], [8]. In this study we describe the identification of a novel splice site mutation in the retinal binding protein-4 (RBP4) gene segregating with recessive retinal degeneration in two affected siblings of a consanguineous Caucasian pedigree and the potential implications of the mutation.

RBP4 is a plasma protein that binds to retinol and facilitates transport of retinol to peripheral tissues [9], [10]. In humans, the circulating RBP4-retinol complex is bound to transthyretin (TTR), a homotetrameric thyroid hormone transport protein. Binding of RBP4 to TTR increases its molecular mass thereby preventing its removal by glomerular filtration [9], whereas, lack of TTR increases urinary excretion of RBP4 [9], [11]. Retinol in peripheral tissues is converted to retinaldehyde, a critical component of the visual cycle [12]. Mice lacking retinol binding protein (RBP) were found to be viable but exhibited impaired vision, demonstrating that retinol binding protein plays an essential role in delivering retinol to the eye [9]. Two teenage sisters identified to be compound heterozygotes for mutations Ile41Asn (rs121918584) and Gly75Asp (rs1218585) in the *RBP4* gene with retinal degeneration has been reported previously by Seelinger et al [20]. One of these siblings had severe acne while the other had iris coloboma. The siblings had no detectable serum retinol binding protein and retinol levels were found to be one sixth of normal levels. These results indicate that RBP4 is important in humans, as RBP is in mice, in delivering retinol to the eye.

In the present study, exome capture and next generation sequencing were carried out to identify the causative mutation in a consanguineous pedigree with two affected members. The progression of retinal pathology was studied in both patients over a period of 17 years. The key feature of the affected siblings was the presence of rod-cone degeneration. While one sibling had ocular colobomas involving both the anterior and posterior segment, the other sibling was without evidence of coloboma. Identification of the mutation in this pedigree establishes the association of RBP4 mutations with severe RD phenotype involving ocular developmental abnormalities.

## Subjects and Methods

### Ethics statement

The studies presented in this manuscript have been approved by the Institutional Review Board of the University of California San Diego. All patient samples were collected with written informed consent. All clinical investigations were conducted according to the principles expressed in the Declaration of Helsinki.

### Subjects

**Figure 1:**
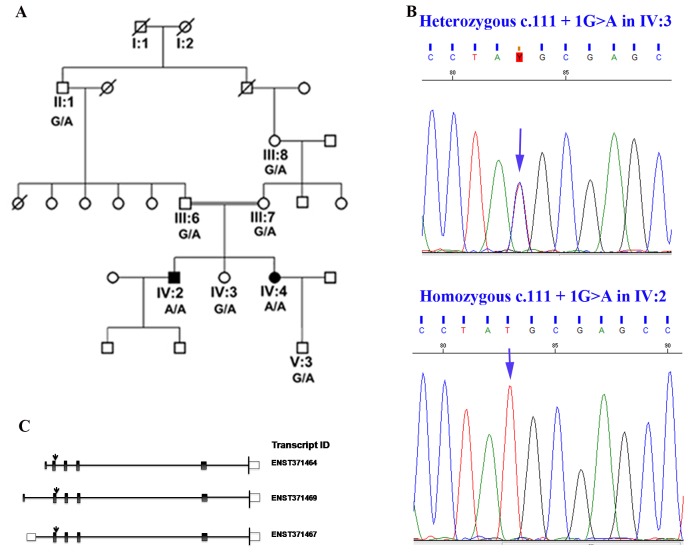
Genetic analysis of a family with retinal degeneration. A. Pedigree of the family with retinal degeneration. Family members that were available for study were genotyped for the RBP4 c.111+1 G>A mutation. B. The reverse complement sequence of the heterozygous subject IV-3 (G/A) and the homozygous subject IV-2 (A/A) are shown. C. Protein encoding transcripts of *RBP4* reported by *Ensembl* (http://uswest.ensembl.org). Three protein coding transcripts are reported by *Ensembl*. Transcripts ENST00000371464 and ENST00000371467 encode the same protein (CCDS 31249). ENST00000371469 does not have a CCDS identifier. The *RBP4* c.111+1 mutation would affect all three of the predicted protein coding transcripts.

Information and blood samples were collected from eight members across five generations of a Caucasian family (Figure 1). Affected subjects IV-2 and IV-4 were examined longitudinally at a seventeen year interval. Informed patient consent and local institutional review board (IRB) approval were obtained for this study which was conducted in accordance with the ethical standards of the 1964 Declaration of Helsinki.

### Ophthalmic evaluation

Ophthalmologic examination of the two affected siblings (IV-2 and IV-4) included best-corrected visual acuity, kinetic perimetry with a Goldmann perimeter using standardized light targets V4e, I4e and I2e, slit-lamp biomicroscopy, slit lamp photography, fundus examination and photography. Absolute luminance threshold to an 11° diameter achromatic central spot was measured centrally and peripherally (10° and 20°) with a Goldmann–Weekers adaptometer (Haag-Streit, Berne, Switzerland) following 45 min of dark adaptation

Full-field electroretinograms (ERGs) were recorded following the standards of the International Society for Clinical Electrophysiology of Vision [14] and using Burian–Allen bipolar contact lens electrodes. An Ag/AgCl electrode was placed on the forehead as the ground. Prior to ERG recording, pupils were dilated fully with topical tropicamide (1%) and phenylephrine (2.5%), and the patient was dark-adapted for 30 min. Scotopic rod-driven responses were recorded first, followed by cone-driven photopic single flash and 30 Hz flicker stimuli.

### Exome caputure and sequencing

All exons in the genome, “exome,” of two affected and one unaffected siblings (IV-2, IV-3 and IV-4) were captured using NimbleGen SeqCap EX exome V1 probes (Roche NimbleGen, Madison, WI) and sequenced using the Genome analyzer GAII sequencer (Illumina, San Diego, CA) following manufacturers' protocols.

Paired end (2×100 bases) DNA sequence reads that passed the quality control were mapped to the human reference genome build hg19 using Bowtie (http://bowtie-bio.sourceforge.net/). Variant calling and annotation were performed using SAMtools (http://samtools.sourceforge.net/) and SeattleSeq (http://snp.gs.washington.edu/SeattleSeqAnnotation/), respectively [15]. Only variants mapping to a single site were further analyzed. Sequence variants that were homozygous in the two affected siblings and heterozygous or absent in the unaffected sibling were identified and the allele frequency of each variant was obtained from dbSNP version 132 reference database (or labeled as novel if not present). Those variants present in genes implicated in retinal diseases were selected for segregation analysis using standard polymerase chain reaction (PCR) followed by dideoxy sequencing as described earlier [16].

### Segregation analysis of the RBP4 c.111+1G>A mutation

Primers that specifically amplify *RBP4* exon 2 (Fwd: 5′-GAT TCC TGG GCA AGA TGA AG-3′, Rev:5′- AAG GAT GAC AGC AGG GGT TT-3′) were used for amplification and sequencing. All available members of the pedigree and 100 ethnicity matched control individuals were screened for the RBP4 c.111+1G>A mutation.

### Extraction and quantification of serum retinol by High-Perormance Liquid chromatography (HPLC)

Serum retinol was assayed by reverse phase chromatography as described previously [17]. Aliquots (50 µL) of serum from three siblings in the pedigree (IV-2, IV-3 and IV-4) and a positive control sample (consisting of 49.8±0.4 µg/dL retinol) were mixed with 20 ng of retinyl acetate (Sigma Chemical Co., St. Louis, MO) in 50 µL ethanol (1∶1, vol∶vol), and extracted twice with 0.5 mL hexane. The retinol containing hexane extracts were pooled and evaporated to dryness under a gentle stream of nitrogen. The residue was dissolved in 100 µL of methanol and 20 µL of the retinol sample was analyzed by HPLC (Thermo Scientific, San Jose, CA), using a 150×4.6 mm Microbondapack C8 (5 µm) column (Millipore). Separation was achieved by using a mobile phase consisting of methanol∶water (95∶5 V/V) at a flow rate of 1.0 mL/min. Retinol was monitored at 326 nm and quantified by comparing the area under the curve with that of retinol (Sigma Chemical Co., St. Louis, MO) analyzed along with the patient samples. The recovery of retinyl acetate was >95%.

### Immunoblotting analysis

Serum RBP4 and TTR protein levels were assessed by immunoblotting as described previously using rabbit polyclonal antibodies to human RBP4 protein (Cat#ab64194; Abcam, Cambridge, MA) and human TTR developed by Raghu et al. [18], [19]. Briefly, the samples were separated by SDS 10%-PAGE and electro-blotted onto nitrocellulose membrane. The membranes were blocked in 5% non-fat dry milk and probed with rabbit anti-human RBP4 or TTR antibody (1 µg, overnight at 4°C) followed by 1 h treatment with goat anti-rabbit IgG-HRP(1∶5000, Pierce). The blots were visualized using SuperSignal® West Pico kit (Pierce Scientific, Rockford,IL).

## Results

### Pedigree

Both affected siblings had the same parents who were related as second-cousins (Figure 1). Each parent harbored the *RBP4* mutation in heterozygous fashion, as did their respective parent (II:1 and III:8) from the family common relationship. Neither of the parents nor their respective parents were examined, but all reported normal vision and no ocular abnormalities. An unaffected female sibling (IV-3) reported having normal vision and no ocular abnormalities.

### Patient IV-2 Phenotype

The affected male (IV-2), was evaluated twice, at age 46 years and again at age 63. He reported a lifelong history of impaired night vision and loss of visual acuity from childhood and a history of hypercholesterolemia. He had surgery for a patent ductus arteriosus at age 46.

**Figure 2:**
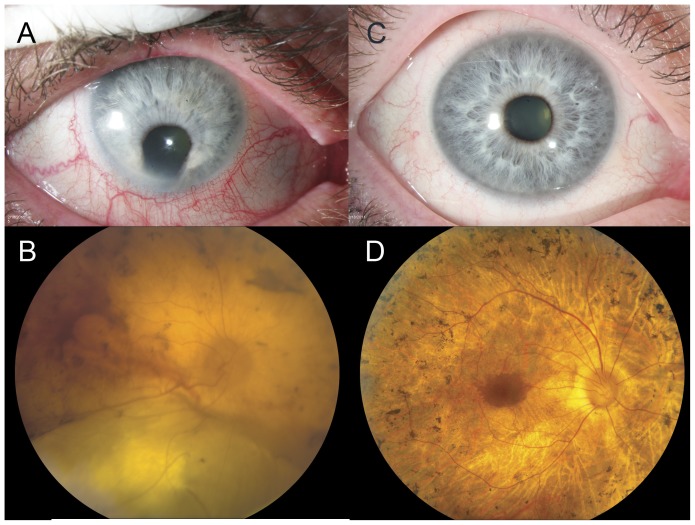
Ocular examination of affected patients IV- 2 at age 63 and IV-4 at age 55. Patient IV-2 exhibited anterior segment dysgenesis with a small cornea, inferiorly displaced pupil, and inferior coloboma of the iris with corneal limbal neovascularization (panel A). The fundus shows an inferior coloboma of the retina and choroid and severe chorioretinal atrophy (panel B). Patient IV-4 has normal anterior segments coloboma or other abnormalities (panel C). The fundus in both eyes was similar with widespread retinal degeneration comprising extensive peripheral retinal atrophy, “bone spicule” pigmentation throughout except for the central-most macula, and markedly attenuated retinal arterioles (panel D).

Ocular examination revealed anterior segment dysgenesis with a small cornea (9 mm), inferiorly displaced pupil, and inferior coloboma of the iris (Figure 2A). He also had evidence of conjunctival and corneal limbal neovascularization, which appeared to be secondary to exposure. The view of the posterior pole was limited due to the presence of significant nuclear sclerotic cataract. Fundus examination revealed inferior coloboma of the retina and choroid (Figure 2B) in addition to the retinal degeneration. At age 46, his vision was hand motion OD and light perception OS. Seventeen years later at age 63 his vision was further reduced to light perception in both eyes. Because of the extent of retinal degeneration, his dark adaptation thresholds, visual fields and ERGs could not be recorded at either visit.

### Patient IV-4 Phenotype

The affected female (IV-4) was evaluated at age 38 years and again at age 55. She reported peripheral vision abnormalities beginning at age 10. Other medical problems include: hypercholesterolemia, acne beginning at a young age and extending into her 50 s, and spine surgery for osteoarthritis. Neither she nor her affected brother had signs of xerophthalmia, keratomalacia, or Bitot's spots.

Her ocular exams revealed normal anterior segments without evidence of coloboma or other abnormality (Figure 2C). Natural lenses were present in both eyes. Her acuities at age 38 years were 20/20 OD and 20/60 OS. Seventeen years later at age 55, her acuities had diminished to 20/32 OD and only hand motion OS. Fundus evaluation revealed evidence of retinal degeneration in both eyes with peripheral retinal atrophy, intraretinal “bone spicule” pigmentation and attenuated retinal arterioles. An island relatively intact retina remained in the central few degrees of the macula surrounding the fovea (Figure 2D).

**Figure 3:**
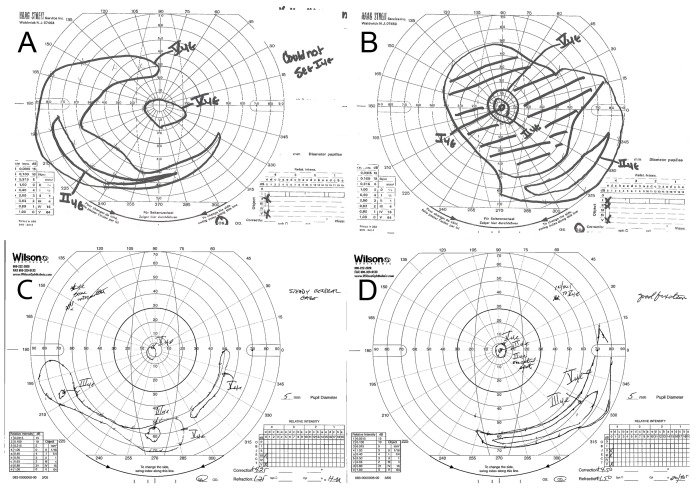
Goldman Visual Field (GVF) of the patient IV-4 at age 38 and at age 55. GVF demonstrated progressive loss from retinal disease. At age 38, both eyes showed a central “tunnel” vision of 15 degrees and large peripheral temporal islands OU (panel A and B), but by age 55, these had constricted to less than 5 degrees, with a small temporal islands for both eyes (panel C and D).

Testing demonstrated severely constricted visual fields OU to less than 5 degrees centrally with a temporal island for both eyes with larger target sizes (Figure 3C and 3D). Evidence of progression in retinal disease is evident across the 17 year interval, as her fields at age 38 were larger centrally and temporally OU (Figures 3A and 3B). By age 55 her dark adapted absolute thresholds centrally were elevated 3–4 log units above rod normal thresholds and were consistent with perception by cones. There were no detectable thresholds at 10 degree and 20 degree eccentricity, consistent with total loss of field sensitivity at these locations on Goldmann perimetry. This was worse than 17 years earlier, as her central threshold then was 2 log units elevated above normal and peripheral sensitivity was detectable at 10 degrees and 20 degrees from fixation thresholds at 3–4 log units elevated over normal rod threshold.

**Figure 4:**
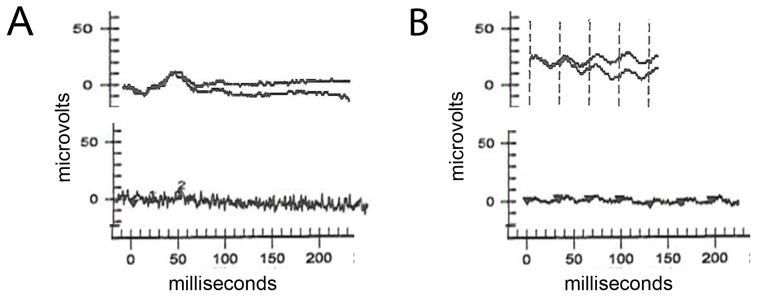
Electroretinogram responses from patient IV-4 with photopic single flash and 30 Hz flicker response stimuli. Both panels show responses at younger age 38 years on top and at 55 years on bottom. The single flash cone responses at age 38 was reduced approximately 80% from normal and was no longer detectable 17 years later (Panel A). The cone flicker response was similarly reduced at age 38 and further diminished by age 55 (Panel B).

ERG scotopic rod-driven responses were not detectable at either visit (data not shown). Cone photopic single flash responses were approximately 80% reduced at age 38 years, and these residual cone responses were further reduced to the level of noise 17 years later (Figure 4A). 30 Hz flicker ERG testing demonstrated a similar decline in this cone elicited response (Figure 4B).

### Exome Analysis

Exome capture and next generation sequencing of patients IV-2 and IV-4 and their unaffected sibling (IV-3) identified ∼8,800–12,000 known single nucleotide variants (SNV) and 1000–1700 novel SNVs in the three subjects. The recessive pattern of inheritance of retinal abnormalities and consanguinity in the pedigree suggested the potential involvement of a homozygous mutation in causing RD in the study family. Analysis of the sequence variants detected in the exomes of all three siblings identified 49 variants that were homozygous in the two affected siblings and simple heterozygous or wild type in the unaffected sibling in 40 unique genes as possible candidates for disease (Table S1). Of these, only RBP4 was a gene that had been previously reported to be associated with retinal degeneration [20].

### Mutation analysis by dideoxy sequencing

Sequencing analysis of the *RPB4* gene in the DNA of all available members of the pedigree revealed the presence of c.111+1G>A change in the homozygous state in intron 2 in both affected members while the unaffected sibling and both parents have this change in the heterozygous state (Figure 1). Analysis of additional members of the pedigree confirmed segregation of this sequence change with the retinal degeneration phenotype. Analysis of 100 ethnicity matched normal controls with no known history of retinal degeneration did not reveal this change indicating that this variant is not likely to be a common variant in this population. The c.111+1G>A change is predicted to disrupt the splice site resulting in alteration of the RBP4 protein sequence. *In-silico* analysis using the NetGene2 software or GeneSplicer software did not identify additional splice sites in the mutant *RBP4* sequence.

### Serum RBP4 protein expression

**Figure 5:**
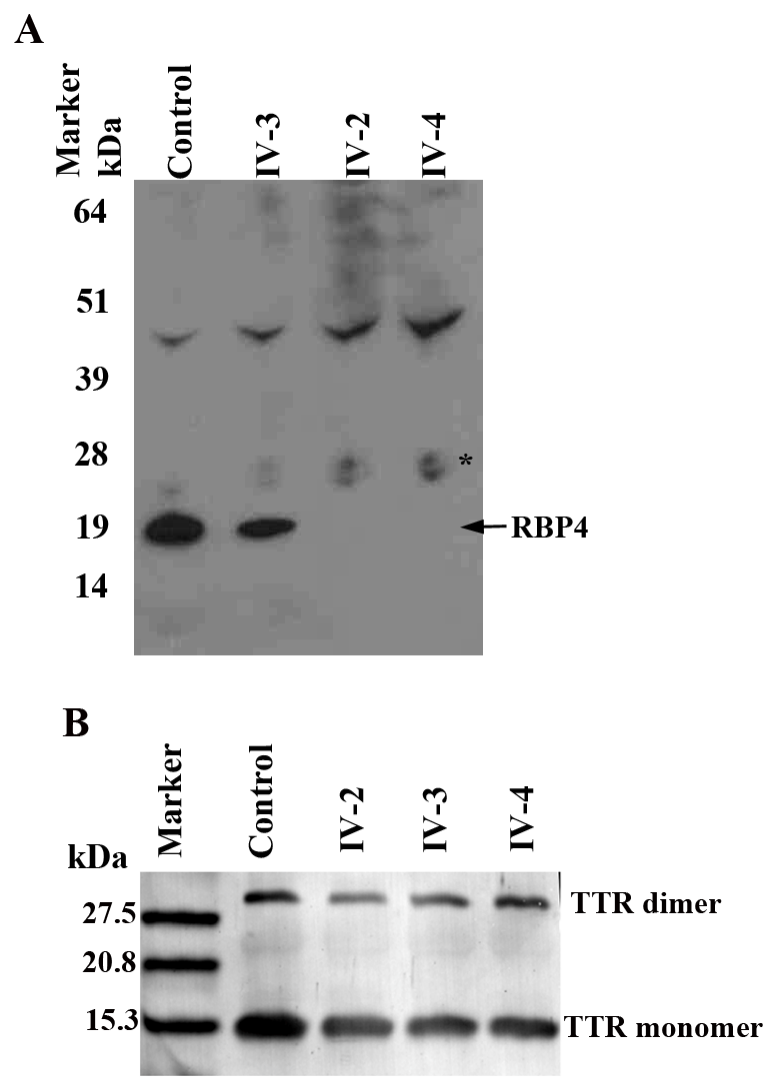
Serum RBP4 and transthyretin (TTR) levels in affected and unaffected family members. Serum RBP4 and TTR levels were examined by western blot analysis as described in Methods. A) The patients, IV-2 and IV-4 had undetectable levels of wild type RBP4 in contrast to their unaffected sibling and the control. A faint band was observed in the region of predicted mutant RBP4 in both patients and their unaffected sibling (Asterisk). B) TTR levels (TTR monomer (15 kDa) and dimer (30 kDa) were indistinguishable between affected and unaffected subjects.

Western blot analysis was performed to determine the effect of the RBP4 c.111+1G>A mutation on RBP4 serum protein levels (Figure 5, top panel). Antibodies to the RBP4 detected the presence of a single immunoreactive band corresponding to the RBP4 protein (∼23 kDa) in the control serum and in the serum of the unaffected sibling (IV-3 in Figure 1). The immunoreactive band corresponding to RBP4 protein was not detected in the serum of the affected patients (Figure 5, top panel, lanes IV-2, IV-4). If the mutation caused the in frame inclusion of 37 amino acids predicted to be encoded by intron 2, the resulting mutant protein would be 26.6 kDa. We observe a faint band of ∼26 kDa in both affected siblings and the unaffected sibling which may represent low levels of the mutant protein (Figure 5: Asterisk). Absence of the wild type RBP4 protein band in IV-2 and IV-4 indicates that the homozygous splice site change affects splicing of the *RBP4* gene and stability of the mutant RNA or protein.

### Serum Transtheyretin (TTR) protein expression

Since absence of transthyretin would also contribute to loss of RBP4 from serum, TTR levels were examined in patients with and without the RBP4 c.111+1G>A mutation by western blot analysis. Presence of immunoreactive bands corresponding to TTR 15 kDa monomer and 30 kDa dimer protein were detected in the control, unaffected sibling, and in both patients (Figure 5b, lanes 1–5) indicating the presence of TTR in patients homozygous for the RBP4 c.111+1 G>A mutation.

### Serum retinol levels

Since absence of RBP4 is expected to cause a reduction in serum retinol levels, we examined the effect of the RBP4 c.111+1G>A mutation on serum retinol levels (Table 1). Retinol was undetectable in the RD affected subjects, while the levels were 49.8±0.4 µg/dL in the control and 30±0.7 µg/dL in the unaffected sister.

**Table 1:** Levels of serum retinol in siblings with the RBP4 c.111+1 G>A change.

Sample (c.111+1 G>A)	Retinol (µg/dL)
Control (Wild type)	49.8±0.4
IV-2 (Homozygous)	undetectable
IV-3 (Heterozygous)	30.0±0.7
IV-4 (Homozygous)	undetectable

## Discussion

Exome sequencing of three members of a consanguineous pedigree identified a novel homozygous *RBP4* mutation in two members with retinal degeneration. Analysis of RBP4, TTR and levels of retinol in serum further established the effect of c.111+1G>A mutation and its association with the retinal degeneration phenotype observed in this pedigree.

The novel mutation in the *RBP4* gene is predicted to disrupt a donor splice site and would affect the mature proteins translated from the three primary protein coding transcripts (ENST00000371464, ENST00000371469, ENST00000371467) (Figure 1c). The RBP4 protein (CCDS 3149) has 201 amino acids with a signal peptide (1–16 aa), retinol binding and lipocalin domains [21]. The predicted mutant RPB4 protein would retain an additional 37 N-terminal amino acids after the signal peptide caused by the in frame inclusion of intron 2, resulting in a total of 238 amino acids. *In silico* analysis did not predict the presence of a cryptic splice site that would result in a frame-shifted and truncated protein. A 238 aa mutant RPB4 protein, if stable, would retain both lipocalin and retinol binding domain sequences and the signal peptide. Western blot analysis of the serum of patients did not reveal the presence of immunopositive band corresponding to the wild type protein, but detected a faint band in the region of a predicted RBP4 mutant protein. These results demonstrate that the *RBP4* mutation causes either RNA or protein instability. RBP4 is the carrier of retinol in human plasma and is involved in transport of retinol from liver to peripheral tissue including the retina [10]. Lack of RBP4 is expected to affect the levels of serum retinol [9], [13]. Consistent with this, the levels of serum retinol were undetectable in both patients with the *RBP4* mutation as compared to their unaffected sister and a normal control. Increased urinary excretion of RBP-retinol complex also occurs with absence of TTR [11] or by blocking the RBP-TTR interaction [22], leading to reduced serum retinol levels [10], [11], [22]. However, the TTR levels of patients with RBP4 c.111+1 G>A mutation and unaffected sibling remained similar, implying that reduced RBP4 levels in both affected siblings is not due to a lack of TTR. Presence of TTR, absence of RBP4 and significantly lowered levels of retinol in both patients with the c.111+1G>A mutation indicate that this mutation causes the loss of functional RBP4 protein and, as a consequence, serum retinol deficiency.

Lack of normal levels of retinol impairs the visual cycle resulting in night blindness at early stages and prolonged deficiency may lead to retinal degeneration [12], [23]. Non-ocular symptoms of retinol deficiency may include dry skin, increased susceptibility to infection and acne [24], [25]. Furthermore it is well established that vitamin A plays a key role in embryonic development including optic fissure closure [26]. Patient IV-2 had prominent developmental abnormalities including patent ductus arteriosus, choroidal and iris colobomas. However, his sister IV-4 did not present signs of developmental abnormalities, but she had acne even in the 5^th^ decade. These indicate variation in the severity of the retinal degeneration phenotype, developmental abnormalities and non-ocular abnormalities in these two siblings with the RBP4 c.111+1G>A mutation. This variation could be due to either genetic modifiers or nutritional factors such as maternal or dietary vitamin A levels during fetal development and adulthood respectively. Lack of other classical symptoms of vitamin A deficiency including xerophthalmia, keratomalacia, or Bitot's spots in these patients suggest that some dietary vitamin A is being delivered to organs independent of RBP4, but RBP4 is required to deliver sufficient levels of vitamin A to prevent retinal degeneration. Therefore, measuring serum RBP4, TTR and retinol levels may be a useful way to identify RD patients with mutations in genes that affect vitamin A levels.

Ocular colobomas associated with other abnormalities have been reported [27]. Although the retinal pathology associated with *RBP4* mutations is indistinguishable from the recessive RD phenotype due to mutations in other genes, the additional presence of acne or developmental abnormalities is unique to patients with *RPB4* associated RD [3].

The retinal degeneration associated with the *RBP4* homozygous splice site mutation in our two siblings progressed significantly over a 17-year interval between our examinations in middle age. These patients reported that their vision loss was substantially less severe during their teenage years, but that it became quite severe in their 4^th^ and 5^th^ decade such that one had essentially no remaining functional vision by age 45 years. The only other individuals reported with *RBP4* associated RD were two siblings studied by Seelinger et al, who were 13 and 17 years old at the time of the study and had less profound phenotypic changes suggesting the progressive nature of retinal disease caused by *RBP4* mutations [20]. Our patients (IV-2 and IV-4) reported using high dose vitamin A supplementation during the 17 years between examinations. During this time their vision loss progressed significantly but supplementation may have contributed to the lack of development of other symptoms of vitamin A deficiency including Bitot spots.

The broad genotypic and phenotypic heterogeneity observed in retinal dystrophies with the involvement of well over 150 known genes underlying cases of recessive RD clearly indicate the need for employing an efficient, cost-effective genome wide mutation screening protocol. Currently, the availability of tools to sequence the exome, identify variants and determine their effect on the protein makes exome analysis the primary choice to identify causative mutations. This methodology is suitable to screen for mutations in known and novel genes even in smaller pedigrees such as the one presented in this study.

In summary, exome sequencing and analysis of variants in three siblings from a consanguineous pedigree revealed that a novel homozygous *RBP4* splice site mutation is associated with RD. The RD phenotype associated with the *RBP4* mutation is progressive and results in severe vision loss by middle age. RBP4 RD was also associated with the presence of developmental abnormalities and severe acne in these patients and may be a hallmark of RBP4 associated RD. Analysis of serum vitamin A levels and serum RPB4 may serve as a cost effective initial screening method in determining the potential involvement of RBP4 in patients with retinal degeneration, specifically those with ocular colobomas.

## Supporting Information

Table S1 **SNPs that are homozygous in both affected siblings and heterozygous or absent in the unaffected sibling.**
(DOC)
