# Supplementary material for: Exome Analysis Identified a Novel Mutation in the RBP4 Gene in a Consanguineous Pedigree with Retinal Dystrophy and Developmental Abnormalities
Source: PLoS One. 2012 Nov 26;7(11):e50205. doi: 10.1371/journal.pone.0050205 (PMC3506607; doi:10.1371/journal.pone.0050205)
Supplement: Table S1 — SNPs that are homozygous in both affected siblings and heterozygous or absent in the unaffected sibling. (DOC) [file pone.0050205.s001.doc]

| **Chromosome** | **Position** | **Reference Base** | **Observed Genotype** | **Amino Acid Change** | **rs SNP ID** | **Gene ID** | **PolyPhen Prediction** |
| --- | --- | --- | --- | --- | --- | --- | --- |
| 1 | 13695685 | G | A/A | NC | NA | PRAMEF19 | benign |
| 1 | 13696022 | A | G/G | NC | NA | PRAMEF19 | benign |
| 1 | 144619346 | G | A/A | NC | 4649563 | NBPF9 | U |
| 1 | 145359049 | A | G/G | NC | NA | NBPF10 | U |
| 1 | 179504043 | C | G/G | GLN>GLU | 6658180 | C1orf125 | U |
| 1 | 179562740 | G | C/C | ARG>SER | 61310274 | TDRD5 | Prob. damag |
| 2 | 165476253 | A | T/T | PHE>ILE | 61748245 | GRB14 | benign |
| 2 | 179315757 | T | G/G | MET>LEU | 80004625 | PRKRA | Poss.damag |
| 3 | 26258075 | A | G/G | NC | 7430815 | LOC645101 | U |
| 3 | 148562272 | G | A/A | ARG>HIS | NA | CPB1 | U |
| 4 | 71232388 | G | A/A | GLY>ARG | 10031844 | SMR3A | U |
| 4 | 71384537 | C | T/T | ARG>TRP | 35286445 | AMTN | benign |
| 6 | 29911086 | T | C/C | SER>PRO | NA | HLA-A | benign |
| 6 | 31846741 | C | A/A | ARG>LEU | NA | SLC44A4 | benign |
| 6 | 32489856 | C | A/A | ASP>TYR | 77853982 | HLA-DRB5 | benign |
| 6 | 33048466 | G | T/T | GLY>stop | NA | HLA-DPB1 | U |
| 6 | 33048602 | C | A/A | ALA>GLU | NA | HLA-DPB1 | benign |
| 6 | 33048606 | G | C/C | GLU>ASP | NA | HLA-DPB1 | benign |
| 6 | 33048663 | G | A/A | MET>ILE | NA | HLA-DPB1 | benign |
| 6 | 33141280 | G | A/A | PRO>LEU | NA | COL11A2 | Prob.damag |
| 6 | 38729511 | T | C/C | TYR>HIS | 61748600 | DNAH8 | Prob.damag |
| 6 | 38746176 | G | A/A | ALA>THR | 61748601 | DNAH8 | benign |
| 6 | 150343148 | A | C/C | LEU>ARG | 78563624 | RAET1L | benign |
| 7 | 6013049 | C | G/G | GLY>ALA | NA | PMS2 | benign |
| 7 | 7118264 | A | G/G | NC | 13229689 | LOC100131257 | Prob.damag |
| 7 | 7118900 | C | T/T | NC | 28609720 | LOC100131257 | Prob.damag |
| 7 | 44047066 | T | C/C | CYS>ARG | 78424385 | SPDYE1 | Poss.damag |
| 8 | 11188743 | C | T/T | ALA>VAL | 13260331 | AMAC1L2 | Poss.damag |
| 8 | 11188752 | G | A/A | GLY>ASP | 6990563 | AMAC1L2 | Prob.damag |
| 9 | 7799653 | G | T/T | PRO>THR | 1127430 | C9orf123 | benign |
| 10 | 13214753 | G | C/C | ALA>PRO | 34630110 | MCM10 | benign |
| 10 | 51225970 | T | C/C | ASN>ASP | NA | PARG,AGAP8 | U |
| 10 | 72517830 | G | A/A | SER>ASN | 10999516 | ADAMTS14 | benign |
| 10 | 74896664 | T | C/C | ASN>SER | 36152134 | ECD | benign |
| 10 | 75406912 | G | T/T | SER>TYR | 34163229 | SYNPO2L | Poss.damag |
| 10 | 75442543 | C | T/T | GLU>LYS | NA | AGAP5 | U |
| 10 | 95360674 | C | T/T | NC | NA | **RBP4** | U |
| 10 | 97687049 | G | C/C | NC | 41291582 | C10orf131 | U |
| 11 | 5141980 | A | G/G | CYS>ARG | 4426129 | OR52A4 | U |
| 11 | 7110548 | C | T/T | ALA>VAL | 11041170 | RBMXL2 | benign |
| 12 | 11244194 | T | C/C | NC | 71443637 | PRH1 | benign |
| 12 | 11244725 | C | G/G | NC | 68157013 | PRH1 | benign |
| 13 | 25591842 | G | T/T | NC | 4328299 | LOC100133284 | Prob.damag |
| 15 | 23610192 | G | A/A | NC | NA | LOC653061 | U |
| 15 | 74328141 | G | T/T | NC | 743581 | PML | benign |
| 15 | 79054900 | C | G/G | GLY>ALA | 7495616 | ADAMTS7 | benign |
| X | 153035798 | G | A/A | VAL>ILE | 2266879 | PLXNB3 | benign |
| X | 153039502 | G | C/C | GLU>ASP | 6643791 | PLXNB3 | benign |
| X | 153629155 | A | G/G | ASN>SER | 12012747 | RPL10 | benign |

**Table S1:** SNPs that are homozygous in both affected siblings and heterozygous or absent in the unaffected sibling.

Abbreviations: NC: Non-coding sequence change; NA: Not available; Prob.damag: Probably damaging; Poss. Damag: Possibly damaging: U: Unknown.
